# The Expanding Role of Alternative Splicing in Vascular Smooth Muscle Cell Plasticity

**DOI:** 10.3390/ijms221910213

**Published:** 2021-09-23

**Authors:** Immanuel D. Green, Renjing Liu, Justin J. L. Wong

**Affiliations:** 1 Epigenetics and RNA Biology Program Centenary Institute, The University of Sydney, Camperdown 2050, Australia; i.green@centenary.org.au; 2 Faculty of Medicine and Health, The University of Sydney, Camperdown 2050, Australia; 3 Vascular Epigenetics Laboratory, Victor Chang Cardiac Research Institute, Darlinghurst 2010, Australia; r.liu@victorchang.edu.au

**Keywords:** alternative splicing, vascular smooth muscle cells, gene expression, cardiovascular disease, splicing-modulating therapies

## Abstract

Vascular smooth muscle cells (VSMCs) display extraordinary phenotypic plasticity. This allows them to differentiate or dedifferentiate, depending on environmental cues. The ability to ‘switch’ between a quiescent contractile phenotype to a highly proliferative synthetic state renders VSMCs as primary mediators of vascular repair and remodelling. When their plasticity is pathological, it can lead to cardiovascular diseases such as atherosclerosis and restenosis. Coinciding with significant technological and conceptual innovations in RNA biology, there has been a growing focus on the role of alternative splicing in VSMC gene expression regulation. Herein, we review how alternative splicing and its regulatory factors are involved in generating protein diversity and altering gene expression levels in VSMC plasticity. Moreover, we explore how recent advancements in the development of splicing-modulating therapies may be applied to VSMC-related pathologies.

## 1. Introduction

The ability for mature cells to dedifferentiate is a rare phenomenon in normal physiology. Once a cell commits to a specific fate or identity, there is a greatly limited capacity for this developmental state to be reversed. This process ensures appropriate somatic cell generation, development and function. However, vascular smooth muscle cells (VSMCs) are one exception. They display extraordinary phenotypic plasticity giving them the ability to differentiate or dedifferentiate depending on environmental cues. A prime example of this phenomenon occurs following damage to blood vessels, wherein differentiated VSMCs respond by dedifferentiating, replicating and migrating to the site of injury to initiate repair. Similarly, angiogenesis, the formation of nascent blood vessels, is characterised by highly plastic VSMCs which interact with endothelial cells to produce new vascular networks. As such, this ability to ‘switch’ between a quiescent contractile phenotype to a highly proliferative synthetic state renders VSMCs as primary mediators of vascular repair and remodelling [1,2,3,4,5]. 

**Figure 1:**
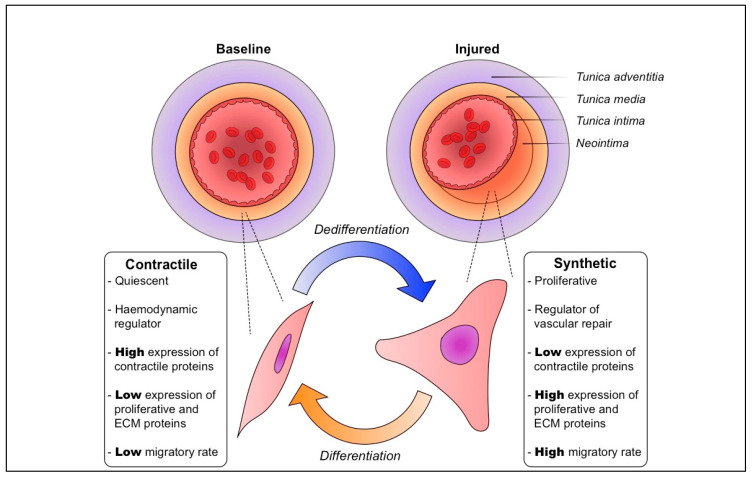
Physiological vascular smooth muscle cell plasticity is vital for vascular repair and homeostasis. At baseline, the tunica media of blood vessels is kept at an appropriate thickness, and vascular smooth muscle cells (VSMCs) have a quiescent, non-migratory contractile phenotype. Following vascular injury, these VSMCs are induced to dedifferentiate to a proliferative, migratory synthetic phenotype. Their growth and increased production of extracellular matrix (ECM) components result in a substantial thickening of the media and formation of a neointima. Followimg vascular repair and reestablishment of homeostasis, synthetic VSMCs are gradually induced to differentiate back to the contractile phenotype.

Differentiated VSMCs have a characteristic contractile phenotype under physiological conditions. Comprising the *tunica media*, these VSMCs control local haemodynamics via coordinated contraction. In arteries and in culture, differentiated VSMCs are enriched with pro-contractile proteins, such as smooth muscle alpha actin 2 (ACTA2) and transgelin (TAGLN), and Ca^2+^ ion channels and signalling factors [1,6]. They express low levels of proliferative and extracellular matrix (ECM) proteins, and are quiescent and largely non-migratory (Figure 1). The contractile state is juxtaposed by the dedifferentiated or synthetic phenotype. Differentiated VSMCs undergo dedifferentiation in response to a deluge of stimulating factors consequent to injury to the vasculature. These include greatly altered local haemodynamics, biomechanical stress, growth factors, cytokines, inflammatory cell mediators, ECM, lipids and reactive oxygen species [7,8,9]. 

VSMC phenotypic plasticity is of major importance after mechanical injury to an artery, particularly during angioplasty and/or stenting. This results in neointimal thickening, the formation of a new layer adjacent to the media (Figure 1) [3,10,11,12]. In these conditions, VSMCs make up the bulk of a thickened neointima in affected vessels, which severely narrows the lumen [3,4,5]. Neointimal thickening after physical injury involves initial proliferation and migration of VSMCs, and is later dominated by a select few (and in some cases, an individual cell) that undergo clonal expansion to make up the majority of a neointima [3,4,5]. 

VSMC phenotypic switching involves a multitude of processes, from the obvious morphological and functional changes, to more subtle alterations in molecular signalling [1,2,13,14,15,16]. These processes are underpinned by a range of gene expression control mechanisms. Great strides have been made to understand how transcription factors and epigenetics regulate phenotypic plasticity by altering gene expression. Many of these studies have focused on histone modifications and DNA methylation, and have been comprehensively reviewed [15,17]. Coinciding with significant technological and conceptual innovations in RNA biology, there has been a growing focus on co-transcriptional processes that regulate gene expression in VSMCs. In particular, alternative splicing (AS) of mRNAs and its regulatory factors have been investigated in a variety of physiological and pathological VSMC contexts [18,19,20,21,22,23,24,25]. Collectively, these studies have revealed an expanding role of AS and splicing factors in promoting protein diversity and regulating gene expression in VSMC biology. In this review, we highlight the major findings from such works and discuss the implications of AS in healthy and pathological VSMC states. Furthermore, we critically evaluate the possible avenues for modulating AS in VSMCs for therapeutic benefit. 

## 2. Alternative Splicing

During transcription from DNA in eukaryotes, nascent precursor mRNAs first emerge with both protein-coding exons and non-coding introns in their sequence. To form mature mRNA transcripts for later translation into protein, the non-coding introns must be excised via intramolecular splicing. This produces mature mRNAs comprising of only exonic sequences, ready for translation. When mature mRNAs have exons occurring in the same sequence as their corresponding precursor mRNA, this form of splicing is constitutive. Since splicing out introns is an energy-intensive process, increased research, discussion and debate has persisted around the evolution of introns and their conservation in eukaryotic biology. It has been previously argued that introns impose a ‘burden’ on an organism [26,27,28]. It seems eccentric that eukaryotic cells invest such substantial time and energy to maintain introns in the genome, only to splice them out during transcription. As detailed in the later part of this review, it is evident that introns are essential to promote protein diversity via AS, which facilitates tissue-specific functions in eukaryotes [29,30,31,32]. Certain introns also contain regulatory elements that regulate gene expression, and others can serve as precursors of non-coding RNAs with roles in a myriad of biological processes [33,34,35,36,37].

Alternative mRNA splicing is characterised by the differential inclusion or exclusion of exons and introns in transcripts. In contrast to constitutive splicing, AS is responsible for producing many mature mRNA isoforms from a single precursor mRNA. This allows the production of multiple proteins from a single gene, meaning genetic information can be stored and preserved in a more economical fashion. Therefore, AS is understood as an efficient way to generate and maintain biological complexity [38]. Deep transcriptomic and proteomic analyses have shown that approximately 95% of human genes are subject to AS, and is one of the main sources of protein diversity [38,39]. As such, AS plays a central role in regulating cell function, proliferation, survival and differentiation [11].

**Figure 2:**
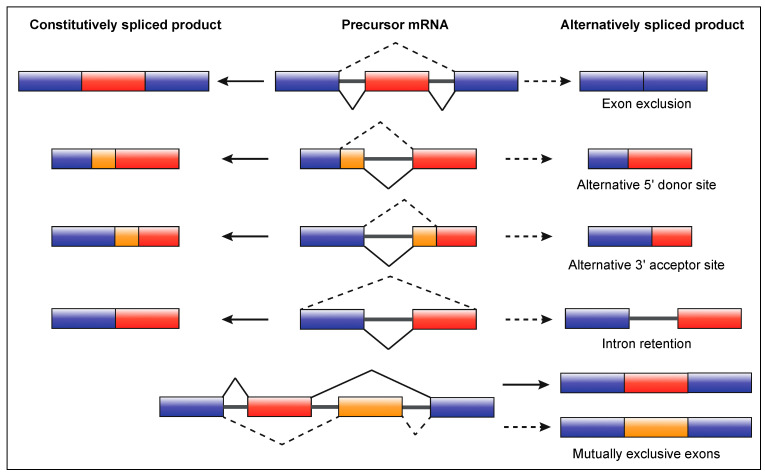
The main types of alternative mRNA splicing. Precursor mRNA can undergo either constitutive or alternative splicing. Constitutive splicing products (left) lack introns and comprise whole exons in the same sequence as the precursor mRNA (middle). Depending on the type of alternative splicing, different products can be yielded (right). Exon exclusion, and alternative 5′ and 3′ splice site selection result in a truncated transcript. Intron retention produces an elongated transcript, as the intron is not excised. Mutually exclusive exons do not occur together in mature mRNA during splicing and produce separate isoforms.

There are five main types of AS which elicit a range of functional consequences, including changes in mRNA stability, localisation and translation (Figure 2). These, in conjunction with the change in mRNA base sequence, can contribute to proteomic diversity or regulate gene expression levels [29,38,40]. Most AS events can result in a truncated mRNA transcript, namely exon exclusion, and alternative 5′ and 3′ splice site selection (Figure 2). Such events, along with mutually exclusive exon splicing, have the potential to significantly increase the repertoire of proteins produced or alter mRNA metabolism. In contrast, intron retention (IR) produces longer transcripts as intronic sequences are preserved in mature mRNA.

Although initially dismissed as an aberration, IR is now established as a key mechanism of gene expression control in many cell types, particularly in the neuronal and haematopoietic lineages and more recently in VSMCs [21,41,42,43,44,45,46,47,48]. IR can result in post-transcriptional gene repression, as many introns contains premature termination codons which facilitate cytoplasmic nonsense-mediated decay [47,49]. Degradation can also occur via the RNA exosome, if intron-retaining transcripts are detained in the nucleus [48,50,51]. Alternatively, following appropriate stimulus, stable nuclear-detained transcripts can accumulate and undergo rapid constitutive splicing to enable a burst of protein synthesis [42,43,44,45,52,53,54].

There are a range of factors which regulate splicing. These include intrinsic sequence features of the genome, such as GC content, relative exon/intron lengths, splice site strength and splicing enhancer or silencer motifs [47,55,56]. Epigenetic changes at the DNA and histone level, such as nucleosome occupancy, chromatin organisation and CpG methylation, have also been strongly implicated [55,57,58]. Another layer of regulation is at the level of differential splicing factor activity and expression levels. These regulatory proteins, along with other RNA binding proteins (RBPs), play essential roles in either enhancing or repressing spliceosome formation at specific sites on nascent mRNA [38]. In the context of AS in VSMC biology, several studies have recently explored the ways specific RBPs and splicing factors influence VSMC function in health and disease [18,19,20,21,22,23,24,25].

## 3. Alternative Splicing in Vascular Smooth Muscle Cell Phenotypic Plasticity

Different forms of AS and specific splicing factors have been found to play distinct roles in regulating VSMC physiological phenotypic plasticity. Transcriptome-wide analyses of different VSMC types and phenotypes have revealed a plethora of global changes in AS, as well as important isoform-switching events in key VSMC genes [21,22,24]. When comparing differentiated and synthetic aortic murine VSMCs, Llorian et al. showed that specific exons were preferentially included in different genes relevant to each phenotype. For example, in differentiated VSMCs, selected exons in genes involved in calcium channels, mobilisation and signalling were found to be significantly included [21]. Several mutually exclusive exons were also identified between the two phenotypes, particularly in genes involved in the actin cytoskeleton and smooth muscle contraction [21]. The polypyrimidine tract-binding protein 1 (PTBP1), an RBP involved in splicing, was found to be decreased in differentiated VSMCs. Its knockdown induced altered usage of mutually exclusive exons in specific transcripts, such as *Actn1* and *Tpm1*, promoting a more proliferative VSMC phenotype [21]. Furthermore, many of these exons were confirmed to possess PTBP1-binding motifs in their upstream intron, suggesting that PTBP1 may act to co-transcriptionally repress AS events associated with the differentiated phenotype in non-differentiated cells [21]. 

**Figure 3:**
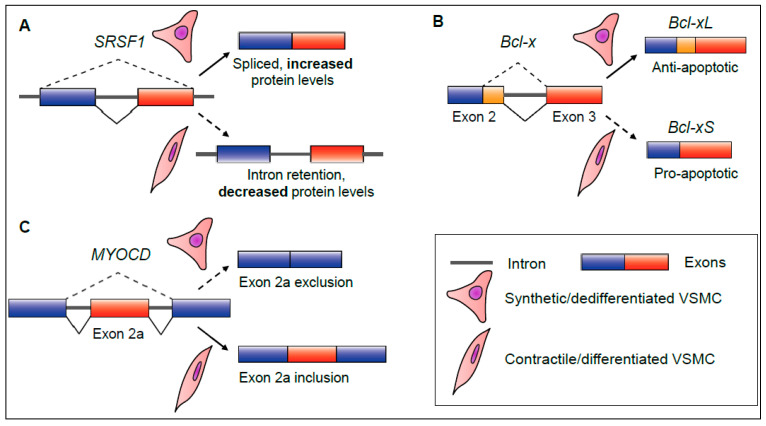
Examples of key splicing events which regulate vascular smooth muscle cell plasticity. (**A**) Serine/arginine-rich splicing factor 1 (*SRSF1*) precursor mRNA undergoes increased intron retention during vascular smooth muscle cell (VSMC) differentiation. This leads to decreased SRSF1 protein expression overall. (**B**) B-cell lymphoma (*Bcl-x*) transcripts undergo alternate splice site selection to produce a long (-xL), anti-apoptotic isoform during VSMC dedifferentiation and cell growth. (**C**) Myocardin (*MYOCD*) transcripts can switch protein isoforms by excluding or including exon 2a during VSMC dedifferentiation or differentiation.

Intron retention was also identified as a significant AS program in differentiating aortic murine VSMCs [21]. The authors referred to IR events as ‘non-productive’, as increased IR was found to be positively correlated with decreased constitutively spliced transcripts or protein levels, consistent with other reports [21,29,42,47]. Additional analyses revealed that a majority of transcripts subject to increased IR during differentiation were found to be nuclear detained, and coded for a range of essential splicing factors, including serine/arginine-rich splicing factor 1 (SRSF1) and U2 small nuclear ribonucleoprotein A (SNRPA1) [21]. Although the exact pathways via which these splicing factors are affected by nucleus-specific IR was not established in this study, there are two main possibilities. These intron-retaining transcripts may be subject to degradation via the RNA exosome, in a similar manner to neuronal precursor cells [48,50,51]. Another possibility is that these transcripts may be sequestered in the nucleus and remain for concerted splicing following an activating stimulus, similar to what occurs during neuronal or macrophage activation [42,43]. This temporary downregulation of splicing factor expression via IR may be important to establish a quiescent, differentiated VSMC phenotype (Figure 3A). Notably, other related SR splicing factor transcripts, Srsf6 and Srsf7, were additionally found to be downregulated via inclusion of ‘poison’ exons containing premature termination codons, making them targets for nonsense-mediated decay [21]. Due to their plasticity, Llorian and colleagues also suggested that this phenomenon may be readily reversible during dedifferentiation [21]. This hypothesis is consistent with the independent finding that functional SRSF1 splicing factor expression is increased in dedifferentiated, proliferative VSMCs [24].

SRSF1 is not only involved in splicing, but a critical regulator of genomic stability, cell development and proliferation [24]. SRSF1 was highly expressed following in vivo vascular injury, and consequent initiation of neointimal hyperplasia. Perturbing its expression in VSMCs strongly inhibited this response [24]. Using human aortic VSMCs, Xie and colleagues showed that SRSF1 was involved in controlling the expression of tumour protein p53 (*TP53*), a primary regulator of cell growth [24]. A truncated TP53 isoform (Δ133p53), was found to be concomitantly upregulated in SRSF-expressing neoplastic VSMCs [24]. Further analyses revealed that this Δ133p53 isoform interacts with the EGR1 transcription factor and activates KLF5 expression, a well-known promoter of VSMC proliferation [1,2,24]. Although the expression of the Δ133p53 isoform has been previously shown to occur via alternative promoter usage, it is as yet unknown whether SRSF1 is implicated in its production via a splicing-independent or splicing-dependent mechanism in VSMCs [24]. This certainly merits further investigation [59]. However, Xie et al. confirmed that SRSF1 modulates global antiapoptotic splicing events in proliferative VSMCs in vitro and in vivo [24]. SRSF1 coordinates the AS of *Bcl-x* transcripts, resulting in a predominance of Bcl-xL proteins, the long anti-apoptotic counterpart to the short pro-apoptotic Bcl-xS isoform (Figure 3B). This comprehensive study demonstrated how the SRSF1 splicing factor promotes VSMC cell growth, and inhibits apoptosis, leading to neointimal hyperplasia [24].

Other RBPs and splicing factors have also been characterised in VSMC phenotypic switching. The RBP quaking (QKI) was found to play a myriad of roles during both VSMC differentiation and proliferation [18,21,23]. Earlier work by van der Veer and colleagues established QKI as a central regulator of in vivo VSMC dedifferentiation following vascular injury [23]. QKI localises to the spliceosome and coordinates the AS of myocardin (*Myocd*), an essential coactivator of the VSMC transcription factor *Srf*. This activity alters the balance between different *Myocd* isoforms, which interact with other transcription factors, leading to the neointimal hyperplastic response by VSMCs. Specifically, QKI excludes *Myocd* exon 2a during dedifferentiation [23]. Notably, A newly identified splicing factor, RBPMS, actively includes exon 2a in *Myocd* transcripts to promote differentiation in vitro [22] (Figure 3C). Llorian et al., expanded upon this area of research by identifying QKI-related binding motifs enriched downstream of differentially spliced exons in other VSMC genes involved in in vitro phenotypic switching [21]. Adding another level of complexity to QKI-mediated AS, QKI itself can be regulated by AS [18,23]. During in vitro differentiation of murine induced pluripotent stem cells (iPSCs) into VSMC-like cells, Caines and colleagues determined that isoform 6 of QKI (QKI-6) induces the establishment of a VSMC-typical phenotype [18]. QKI-6 controls the AS of histone deacetylase 7 (*Hdac7*) by binding to its first intron via a QKI-binding motif. This generates an alternate, longer isoform, HDAC7s, which is differentially active to the unspliced HDAC7u isoform [18]. HDAC7s enhances the activation of SRF-MYOCD-regulated transcription of VSMC genes, such as *Tagln* [18].

Taken together, these studies demonstrate the importance of AS in regulating VSMC plasticity. Splicing factors and RBPs, such as PTBP1, SRSF1, QKI and RBPMS are critical for diversifying the VSMC proteome and, in particular, producing isoforms that drive VSMC differentiation or dedifferentiation (Table 1) [18,21,22,23,24]. These findings highlight the immense utility AS provides for cells and tissues to coordinate complex changes in phenotype, and point to a number of directions for future study. Substantial advancements in cell preparation techniques and high throughput transcriptomics have allowed for single-cell RNA-Seq (scRNA-Seq) of VSMCs in healthy vessels [14]. Although Dobnikar and colleagues’ work did not explore AS at the level of individual VSMCs, improvements in computational and scRNA-Seq technologies have made this an achievable path for future research [19,20]. One significant area of interest is understanding how a select few, or an individual VSMC undergo clonal expansion to make up the majority of a neointima after vascular injury [3,4,5]. Elucidating how heterogeneous AS is across subpopulations of VSMCs may further elucidate how splicing factors are involved in the induction of localised phenotypic switching [19].

## 4. Alternative Splicing in Specific Vascular Smooth Muscle Cell Pathologies

VSMCs are heavily implicated at all stages of atherosclerotic progression and restenosis [3,10,11,12]. As research in the VSMC and cardiovascular disease fields has developed over the previous decade, there is now a clearer understanding of how physiological and pathological VSMC phenotypic plasticity are distinct [11]. This certainly extends to the context of AS, as splicing factors and other mediators can play differing roles in a range of VSMC pathologies [19,20,23,25].

Similar to what was observed during in vivo murine intimal hyperplasia, QKI is highly expressed in the VSMCs of human restenotic lesions compared to healthy controls [23]. Restenosis is the reoccurrence of stenosis, the abnormal narrowing of a blood vessel. It is generally associated with mechanical injury to an artery during balloon angioplasty and/or stenting, used to artificially widen and hold open a narrowed or blocked artery to improve blood flow [12]. Restenotic lesions are part of a rapid atherogenic response by VSMCs, characterised by high levels of inflammation. van der Veer and colleagues demonstrated that VSMCs are the main QKI-expressing cell type in restenotic lesions, and that the RBP is localised at inflammatory foci [23]. These findings reinforce the importance of QKI in regulating VSMC plasticity, and especially during restenotic development.

In addition to chronic cardiovascular diseases, like atherosclerosis, VSMCs are also involved in acute pathologies. Acute aortic dissection (AAD) is a sudden and often fatal condition, wherein the innermost intimal layer of the aorta tears, causing a rupture and significant haemodynamic and aortic instability [19]. Long term hypertension (high blood pressure) promotes the development of AAD. Under healthy conditions, VSMCs are highly differentiated, and their contractility confers stable aortic tension and function. However, during the pathogenesis of AAD, VSMCs adopt a more dedifferentiated phenotype, and weaken the aortic wall via production of proteases which degrade and remodel the ECM [19]. Huan et al. developed an in vivo model of AAD in mice and identified DExH-box helicase 9 (DHX9) as a critical regulator of AAD pathogenesis [19]. The nuclear-localised RBP fulfils a variety of DNA and RNA processing functions, and was found to be expressed at low levels in AAD tissues compared to healthy controls [19]. Using scRNA-Seq analyses of DHX9-knockdown primary VSMCs, the authors revealed that over 1000 genes were subject to AS changes, with almost 20% of these showing significant changes in gene expression regulation [19]. Gene ontology analyses showed that many of these affected genes belonged to functional networks related to splicing, VSMC development, migration and contraction [19]. Further analyses confirmed that DHX9 interacts with another RBP, YB-1, which may facilitate *Klf5* AS. This study not only identified a novel pathogenic mechanism for AAD and VSMC dysfunction, but highlighted the utility of scRNA-Seq in studying the role of AS in cardiovascular disease [19].

Strong arterial tension and contraction relies heavily on appropriate Ca^2+^ ion channelling by VSMCs. Ca_V_1.2 calcium channels are essential regulators of Ca^2+^ influx into cells, and their dysfunction can result in altered arterial tension. That is, overactive Ca_V_1.2 channels can result in increased Ca^2+^ influx, causing abnormal arterial tone and contributing to the pathogenesis of hypertension [25]. The RNA binding motif 9 protein (RBM9, also known as Rbfox2) has been shown to regulate AS of *Cacna1c* exons 9* and 33 during neuronal development, by binding to UGCAUG elements [25,60]. *Cacna1c* codes for an essential subunit of Ca_V_1.2. Using a hypertensive model in rats, Zhou and colleagues’ study sought to investigate whether this was also a feature in arterial VSMCs [25]. Compared to controls, Rbfox2 expression was shown to significantly increase in hypertensive arteries. Concomitantly, there was a critical shift in the Ca_V_1.2 isoform profile; *Cacna1c* mRNA was more likely to include exon 9* and exclude exon 33 [25]. Further electrophysiological analyses found that this isoform exclusively affects the Ca_V_1.2 channel kinetics, resulting in hyperpolarisation and changes in activation and inactivation [25]. Rbfox2 was confirmed to play a direct role in regulating Ca_V_1.2 AS, suggesting that the RBP may be a crucial regulator of Ca^2+^-mediated contraction of VSMCs, and the pathogenesis of hypertension [25].

Technological and conceptual advancements in transcriptomics have illuminated a growing role of circular RNAs (circRNAs) in the cardiovascular system and VSMC-related pathologies [20,61,62,63,64]. circRNAs comprise stable loops, often derived from back-splicing events in precursor mRNAs [20,62]. These molecules are involved in a variety of processes which alter gene expression control, including acting as microRNA sponges and interacting with RBPs to alter mRNA splicing [61]. Liu et al. characterised the role of a novel circRNA, circUVRAG, in the pathogenesis of intimal hyperplasia following vein grafts, a common therapy for coronary disease [20]. circUVRAG levels were found to decrease significantly in vein graft tissues, compared to controls, and was associated with increased VSMC migration and adhesion [20]. Moreover, *Uvrag* precursor mRNA was co-localised with the brain-specific splicing factor, NOVA1, in VSMC nuclei. This, together with NOVA1 knockdown studies, indicates that NOVA1 could be a key splicing factor in the generation of specific circRNAs in VSMCs [20]. Liu and colleagues speculated that circUVRAG may act to finetune or inhibit excessive thickening of vessels [20]. The exact mechanisms via which NOVA1 and circUVRAG mediate vein graft-induced intimal hyperplasia merits further research.

Collectively, these studies highlight a range of AS events, RBPs and splicing factors, which are key regulators of several VSMC and cardiovascular diseases (Table 2). Although most studies have focused on pathologies characterised by dedifferentiated VSMCs, including atherosclerosis, restenosis, AAD, and vein graft disease [19,23,25], significant progress has been made in understanding those involving abnormally contractile VSMCs [25]. More work in the role of VSMC AS in the pathogenesis of hypertension would be a worthwhile contribution to the cardiovascular field, especially as this condition underlies many important cardiovascular pathologies [19,65]. The identification of specific AS events, RBPs and splicing factors which regulate pathological VSMC plasticity paves the way for further research into effective interventions. Recent advancements in pharmacogenomics and transcriptomics have opened up several strategies for modulating AS in diverse disease states for therapeutic benefit [66,67,68,69]. Exploring how these strategies may be utilised in remedying abnormal VSMC plasticity could produce novel approaches to treating a range of cardiovascular pathologies.

## 5. Perspectives for Therapeutic Modulation of Alternative Splicing in Vascular Smooth Muscle Cells

Over 25 years ago, there was an explosion of high-profile studies investigating the utility of antisense oligonucleotide (ASO) therapies in altering VSMC gene expression in vivo [70,71,72,73,74,75,76]. ASOs are strands of modified nucleotides which, depending on their sequence, can bind to target mRNAs and trigger a range of downstream effects. These include blocking ribosome recruitment to inhibit protein production, altering splicing or polyadenylation, or triggering mRNA decay [66,67,68]. Early studies in VSMCs demonstrated that ASOs could be used to inhibit pathological neointimal hyperplasia by reducing expression of proliferative genes. However, due in part to the technological limitations of the time, this area of research in VSMCs has experienced considerable stasis [66,68,76]. In recent years, there has been notable advancements in ASO research, and several ASO drugs have received regulatory approval from the FDA [69]. Most notable are those successfully utilised in the treatment of Duchenne muscular dystrophy and spinal muscular atrophy, which modulate mRNA splicing [28,29,68]. Although there is greatly renewed potential for ASOs to be utilised in VSMCs in a variety of ways, this review focuses on how targeted splicing modulation may address pathological VSMC plasticity.

Targeted binding of ASOs to splice sites and RBP binding motifs can direct precise and consistent changes in how the spliceosome processes precursor mRNA. Depending on the binding location and interactions with splicing factors or RBPs, AS events including exon exclusion/inclusion or intron retention can be induced [29,68]. As discussed earlier, distinct exon inclusion/exclusion events in key VSMC genes play important roles in promoting either differentiation or dedifferentiation, via isoform switching [18,21,22,23,25]. For example, *MYOCD* exon 2a exclusion is associated with the neointimal hyperplastic response, while its inclusion is associated with VSMC-like differentiation [22,23]. Modulating the splicing of vital regulators of phenotypic switching, like MYOCD or Bcl-x, could inhibit pathological VSMC dedifferentiation in diseases such as atherosclerosis or restenosis.

Small molecular compounds which target core factors in the spliceosome have received growing attention in the field of cancer therapeutics [77,78]. An abnormally high expression of splicing factors has been linked to carcinogenesis, and so, functional inhibitors have been developed to perturb tumour cell growth [77,78]. Drugs which indirectly target the SR family of splicing factors, including SRSF1, by inhibiting upstream kinases have been shown to be effective in preclinical trials [77]. Targeting core splicing factors, like SRSF1, using similar strategies in aberrant VSMC proliferation may thereby inhibit the pathogenesis of atherosclerosis and restenosis [21,24].

However, small molecular compounds have also presented some limitations relating to their selectivity [77,78]. Attempting to pharmacologically target ubiquitously expressed splicing factors in VSMCs may cause undesired impacts on other tissues and cell types. Depending on their design, one advantage that ASOs can present is their high selectivity. Modulating individual AS events in genes predominately expressed by VSMCs may decrease the risk of unwanted pleiotropic effects. This includes possible impacts on the AS and RNA metabolism of cells adjacent to VSMCs, particularly in the endothelium. It is also important to consider the risks of ASOs eliciting unpredicted off-target effects within the target tissues and cells themselves. This can depend on its sequence features and concentration introduced to cells [68,69]. Two such consequences are promiscuous binding activity by ASOs and saturation of endogenous RNA processing pathways, adversely affecting cell function [69]. Precise design and chemical modifications made to the ASO, in conjunction with an appropriate delivery strategy, can reduce such risks [69]. 

Targeting a VSMC splicing event, to treat atherosclerosis or restenosis, requires an ASO to not only be delivered to cells embedded in a dense extracellular matrix, but also effectively shuttled to the nucleus. Early work by Pickering et al. demonstrated that ASOs with sulphur-modified linkages (phosphorothioate oligonucleotides) were stably incorporated into cells within atherosclerotic plaques, and translocatable to the nucleus [76]. However, the overall uptake of the ASO into cells was variable and relatively low [76]. A variety of novel ASO delivery strategies developed since may improve VSMC uptake, such as antibodies, cell-penetrating peptides, lipid particles, exosomes or DNA cages [69].

Owing to their plasticity, an important challenge to modulate AS in VSMCs is their heterogeneity in healthy and diseased vessels [3,14]. Depending on the differing AS and gene expression patterns within a population of VSMCs, an ASO may exert highly variable impacts on the target tissue. However, further understanding of how AS may vary across VSMC populations, and using methods like scRNA-Seq may render this apparent limitation into a worthwhile advantage. Identifying and targeting specific AS events characteristic of a subset of VSMCs, which form the bulk of a neointima, could enhance the selectivity and potency of the ASO therapy.

Although research exploring ASOs in VSMC pathologies has experienced some dormancy, modern innovations have renewed their potential to be possible interventions for cardiovascular diseases [66,68,69]. This area certainly merits further investigation, especially in understanding and addressing the challenges presented by VSMCs’ unique phenotypic plasticity.

## 6. Conclusions

Significant advancements in the research of alternative splicing have revealed its deep and complex role in regulating VSMC plasticity. In both health and disease, AS and its regulatory factors control key events in VSMCs, leading to changes in protein diversity and gene expression levels. In contexts where VSMC plasticity is pathological, an expanding body of evidence has demonstrated how dysregulated AS is a vital feature of severe cardiovascular diseases. More progress in understanding the underlying mechanisms for these events may pave the way for developing suitable therapeutic interventions.

## Figures and Tables

**Table 1:** Summary of primary regulators and outcomes of alternative splicing in vascular smooth muscle cell plasticity.

VSMC Biological Process	Splicing Factor, RBP or AS Event	VSMC-Relevant Transcripts Affected	Reference
Murine aortic VSMC differentiation	Specific exon inclusion events	*Cald1*, *Cacna2d1*—Calcium transport and mobilisation	[21]
Murine aortic VSMC differentiation or proliferation	PTBP1 regulates specific mutually exclusive exon inclusion events	*Actn1*, *Tpm1*—Cytoskeleton and VSMC contraction	[21]
Murine aortic VSMC differentiation	Intron retention coupled with nuclear detention	*Srsf1*, *Snrpa1*, *Sf3b1*, *Rbm3*—Splicing factors and RBPs	[21]
Murine VSMC neointimal hyperplasia and human VSMC proliferation	SRSF1	Δ*133p53*—Cell growth *Bcl-xL*—Cell survival	[24]
Murine VSMC neointimal hyperplasia	QKI regulates a specific exon exclusion event (*Myocd exon 2a*)	*Myocd*—Coactivator of VSMC transcription	[23]
Differentiation of PAC1 VSMCs	RBPMS regulates a specific exon inclusion event (*Myocd exon 2a*)	*Myocd*—Coactivator of VSMC transcription	[22]
Differentiation of murine iPSCs to a VSMC-like phenotype	Alternatively spliced QKI-6 induces a specific exon inclusion event	*Hdac7s*—Coactivator of VSMC transcription via MYOCD-SRF	[18]

VSMC, vascular smooth muscle cell; RBP, RNA binding protein; AS, alternative splicing; PTBP1, polypyrimidine tract-binding protein 1; SRSF1, serine/arginine-rich splicing factor 1; QKI, quaking; RBPMS, RNA binding protein of multiple splicing; iPSCs, induced pluripotent stem cells.

**Table 2:** Summary of primary regulators and outcomes of alternative splicing in vascular smooth muscle cell pathologies.

VSMC Pathology	Splicing Factor, RBP or AS Event	VSMC-Relevant Transcripts Affected	Reference
Human coronary restenosis	QKI	*MYOCD*—Coactivator of VSMC transcription	[23]
Development of acute aortic dissection	DHX9 and YB-1 interact to modulate specific AS events	*Klf5*—VSMC proliferation	[19]
Hypertension	RBM9/Rbfox2 regulates differential inclusion and exclusion events of specific exons (*Cacna1c* exons 9* and 33)	*Cacna1c*—component of Cav1.2 calcium channel	[25]
Vein graft induced intimal hyperplasia	NOVA1 involved in circUVRAG generation from *Uvrag* AS	circUVRAG—cell migration and adhesion	[20]

VSMC, vascular smooth muscle cell; RBP, RNA binding protein; AS, alternative splicing; QKI, quaking; DHX9, DexH-box helicase 9; YB-1, Y box binding protein 1; RBM9/Rbfox 2, RNA binding motif 9; NOVA1, NOVA alternative splicing regulator 1; circUVRAG, circular RNA UVRAG.

## Data Availability

Not applicable.
